# Increased Sensitivity to Inflammatory Pain Induced by Subcutaneous Formalin Injection in Serine Racemase Knock-Out Mice

**DOI:** 10.1371/journal.pone.0105282

**Published:** 2014-08-18

**Authors:** Ayako Tabata-Imai, Ran Inoue, Hisashi Mori

**Affiliations:** Department of Molecular Neuroscience, Graduate School of Medicine and Pharmaceutical Sciences, University of Toyama, Toyama, Japan; Tokyo Metropolitan Institute of Medical Science, Japan

## Abstract

D-Serine, an endogenous coagonist of the *N*-methyl-D-aspartate receptor (NMDAR), is widely distributed in the central nervous system and is synthesized from L-serine by serine racemase (SR). NMDAR plays an important role in pain processing including central sensitization that eventually causes hyperalgesia. To elucidate the roles of D-serine and SR in pain transmission, we evaluated the behavioral changes and spinal nociceptive processing induced by formalin using SR knock-out (KO) mice. We found that SR is mainly distributed in lamina II of the dorsal horn of the spinal cord in wild-type (WT) mice. Although the formalin injected subcutaneously induced the biphasic pain response of licking in SR-KO and WT mice, the time spent on licking was significantly longer in the SR-KO mice during the second phase of the formalin test. The number of neurons immunopositive for c-Fos and phosphorylated extracellular signal-regulated kinase (p-ERK), which are molecular pain markers, in laminae I-II of the ipsilateral dorsal horn was significantly larger in the SR-KO mice. Immunohistochemical staining revealed that the distribution of SR changed from being broad to being concentrated in cell bodies after the formalin injection. On the other hand, the expression level of the cytosolic SR in the ipsilateral dorsal horn significantly decreased. Oral administration of 10 mM D-serine in drinking water for one week cancelled the difference in pain behaviors between WT and SR-KO mice in phase 2 of the formalin test. These findings demonstrate that the SR-KO mice showed increased sensitivity to inflammatory pain and the WT mice showed translocation of SR and decreased SR expression levels after the formalin injection, which suggest a novel antinociceptive mechanism via SR indicating an important role of D-serine in pain transmission.

## Introduction

D-Serine, an endogenous coagonist of the *N*-methyl-D-aspartate receptor (NMDAR), is widely distributed in the central nervous system (CNS) and is synthesized by the biosynthetic enzyme serine racemase (SR) [1], [2], [3]. By binding at the glycine binding site of NMDAR with an agonist, glutamate, D-serine efficiently activates NMDAR [4], [5], [6]. By modulating neurotransmission through NMDAR, D-serine plays important roles under physiological and pathophysiological conditions including synaptic plasticity, learning and memory, and neuronal toxicity [7], [8], [9], [10].

Involvement of NMDAR in pain transmission has been the focus of much research in terms of central sensitization, the process for the establishment of the hyperexcitable state in the spinal cord that leads to enhanced responses to nociceptive stimuli [11]. Under repetitive noxious stimulation, excitatory postsynaptic currents (EPSCs) generated by glutamatergic transmission from nociceptors summate in postsynaptic neurons and activate NMDAR. NMDAR activation causes calcium influx, which modulates synaptic strength that leads to the enhancement of nociceptive transmission and development of hyperalgesia [12], [13]. NMDAR antagonists, on the other hand, abolish or prevent the state of hyperexcitability induced by central sensitization [14]. The NMDAR antagonist ketamine has been applied as an effective analgesic adjunct for postoperative pain in humans [15].

NMDAR is basically composed of NR1 and NR2 (A - D) subunits and forms a heterotetramer between two NR1 subunits and at least one of other NR2 subunits [16]. The NR1 subunits are distributed throughout the gray matter of the spinal cord, whereas the NR2A subunit is concentrated in laminae III and IV and the NR2B subunit in lamina II of the dorsal horn [17], [18]. The NR2B subunit in lamina II, where nociceptive fibers terminate, is involved in neuronal plasticity and pain transmission [19], [20], [21]. The antagonists selective to the NR2B subunits administered intrathecally attenuate the pain response in inflammatory and neuropathic pain models [22], [23]. Because the distribution of D-serine in the brain closely matches that of NMDAR, particularly that containing the NR2B subunit [1], [24], it has been postulated that D-serine is involved in pain transmission.

The lines of evidence of the contribution of D-serine in pain transmission are, however, controversial. In the studies using transgenic mice lacking D-amino acid oxidase (DAO), an enzyme that catalyzes the oxidative deamination of D-amino acids, Wake et al. reported that pain-related responses in the formalin test were augmented [25], whereas Zhao et al. reported that they were attenuated [26]. DAO inhibitors administered intrathecally attenuated and reversed formalin-induced pain-related responses [27]. D-Serine directly applied to the rat dorsal horn increased NMDAR-mediated EPSCs [28]. Intrathecal administration of D-serine facilitated the tail flick reflex in response to thermal stimuli [29], and antagonized the antinociceptive effect of gabapentin [30] and lidocaine [31], or did not affect formalin-induced pain behavior [32].

In this study, we evaluated the behavioral changes and the spinal nociceptive processing in response to the inflammatory pain induced by formalin using SR knock-out (KO) mice as the control to elucidate the role of endogenous D-serine in pain transmission.

## Materials and Methods

### Ethics statement

The animal care and experimental protocol were approved by the Animal Experiment Committee of the University of Toyama (Approval number: A2011 MED-7) and were carried out in accordance with the Guidelines for the Care and Use of Laboratory Animals of the University of Toyama. Tissue sampling was performed under overdosing with sodium pentobarbital or halothane, and all efforts were made to minimize suffering.

### Animals

Generation and genotyping of SR-KO and wild-type (WT) control mice with the pure C57BL/6 genetic background have been reported [33]. Adult (3–5 months) age-matched SR-KO and WT male mice were used in this study. Because diurnal circadian rhythm [34], ambient temperature [35], and acute stress [36], [37] affect the pain threshold, the entire experiment was performed from 10:00 a.m. to 4:00 p.m. at room temperature (RT) between 21.5 and 24.5°C, after acclimatizing the mice to the experimental environment. The experiment was also performed with chemical restrain using the anesthetic halothane before formalin injection.

### Antibodies and reagents

The primary antibodies and staining reagents used in immunohistochemical analysis were as follows: 1) the mouse monoclonal anti-SR antibody (BD Biosciences; 1∶100), 2) the mouse monoclonal anti-NeuN antibody [Chemicon (Millipore); 1∶100], 3) the rabbit anti-c-Fos antibody (Santa Cruz Biotechnology; 1∶5,000), 4) the rabbit anti-p-ERK antibody (Cell Signaling Technology; 1∶500), and 5) the rabbit anti-microtubule-associated protein 2 (anti-MAP2) antibody (Millipore; 1∶100). The secondary antibodies used were Alexa-488- and Alexa-594-labeled species-specific antibodies (Invitrogen; 1∶500). All the primary and secondary antibodies were diluted with 1% bovine serum albumin (BSA) in phosphate-buffered saline (PBS). FITC-conjugated isolectin IB4 (Vector Laboratories; 1∶50 in PBS) was used for staining lamina II of the dorsal horn. For blocking nonspecific signals in the prepared tissue sections, 10% donkey normal serum in PBS was used. For p-ERK staining, 0.3% Triton-X 100 was added to the blocking and antibody-diluting solutions.

The primary antibodies used in western blot analysis were 1) the rabbit anti-SR antibody (1∶500) [38], 2) the rabbit anti-actin antibody (Santa Cruz Biotechnology; 1∶5,000), and 3) the rabbit anti-transferrin receptor (anti-TfR) antibody (Millipore; 1∶1,000). Horseradish peroxidase (HRP)-conjugated anti-rabbit IgG (Bio-Rad; 1∶10,000) was used as the secondary antibody.

### Immunohistochemical analysis

Mice were deeply anesthetized with sodium pentobarbital (100 mg/kg body weight, IP) and transcardially perfused with ice-cold PBS followed by 4% paraformaldehyde in PBS. The mice used in the formalin injection study were euthanized with halothane (4%) after the experiment. Their spinal cords were harvested without perfusion fixation to avoid the possible loss of immunohistochemical signals and postfixed in 4% paraformaldehyde in PBS at 4°C overnight. After cryoprotection with 30% sucrose in 0.1 M phosphate buffer, the lumbar enlargements of the spinal cords were cut and embedded in O.T.C. mounting medium (Tissue-Tek, Sakura Finetek Japan Co., Ltd., Tokyo). The spinal cords were transversally cut into 20-µm-thick sections with a cryostat (LEICA CM1850, Leica Instruments GmbH, Germany) and the sections from L4–L5 were morphologically identified under a light microscope.

Sections for staining with mouse monoclonal antibodies were pretreated with a mouse Ig blocking reagent (Vector Burlingame, CA). Sections for staining with an anti-p-ERK antibody were pretreated by permeabilization with ice-cold methanol and incubation at −20°C for 10 min. After 1 h incubation at RT with the blocking solution, tissue sections were incubated with primary antibodies described above at 4°C overnight. Sections were incubated with secondary antibodies for 1 h at RT. To identify lamina II of the dorsal horn, the sections were incubated with FITC-conjugated IB4 at RT for 1 h. All the sections were counterstained with 4′, 6-diamidino-2-phenylindole (DAPI) for cell nuclear visualization and coverslipped. Images were taken using a confocal laser scanning microscope, Leica TCS-SP5 (Leica Microsystems, Mannheim, Germany).

### Formalin test

Nine WT and eleven SR-KO mice were used for the formalin test. The animals were individually handled for a week before the experiment to acclimatize them to the experimental environment. The formalin test was performed in accordance with the method described by Malmberg and Yaksh [39]. The mice were lightly anesthetized with halothane and 20 µl of 5% formalin solution (formaldehyde solution, Sigma-Aldrich; concentration was adjusted using sterile saline) was injected subcutaneously into the dorsal surface of the left hind paw using a 30-gauge needle. The mice were individually placed in a mirror-backed Plexiglass chamber (25×25×20 cm^3^) for observation. After a brief recovery period (less than 2 min), the mice showed normal motor function, and then pain and other spontaneous nonpain behaviors were observed and video-recorded for 120 min. The durations of pain and nonpain behaviors were analyzed over periods of 1–5 min and 6–10 min, and at 10 min intervals after that until 120 min. The mice were euthanized with halothane (4%) 30 min after the formalin test, and their spinal cords were sampled for further analysis.

For the formalin test after oral administration of D-serine, nine WT and ten SR-KO mice were used. D-Serine (Wako, Japan) was dissolved in water at 10 mM and was administered to the WT and SR-KO mice with drinking water for one week. The formalin test and pain behavioral analysis were performed as described above.

### Behavioral analysis

Mouse behaviors observed during the formalin test were analyzed using the recorded video images. Licking of the formalin-injected paw was considered as a pain behavior [40] and the time spent on licking was visually determined in units of seconds. Other spontaneous behaviors, such as walking (moving forward or backward on four limbs), rearing (standing on hind legs with the stifle extended), grooming, and crouching, were considered to be nonpain behaviors and the duration was determined in units of seconds. Behaviors were analyzed by an experimenter who was blind to the genotype of the mice used in the study.

### Counting of c-Fos- and p-ERK-immunopositive neurons in dorsal horn of mice after formalin test

The spinal cords harvested 30 min after the formalin test were processed for immunohistochemical analysis using the following primary antibodies: mouse monoclonal anti-NeuN, rabbit polyclonal anti-cFos, and rabbit polyclonal anti-p-ERK antibodies. Neurons were identified by staining with the anti-NeuN antibody. All the sections were counterstained with DAPI for cell nuclear visualization.

Five SR-KO and five WT mice were used in this study. Four nonadjacent sections from the L4–L5 lumbar spinal cord from each mouse were randomly selected before the examination of c-Fos and p-ERK immunostaining. The images of c-Fos and p-ERK staining in the dorsal horn ipsilateral to the formalin-injected hind paw were evaluated. When c-Fos or p-ERK staining in a cell was colocalized with nuclear staining (DAPI), the cell was counted as c-Fos- or p-ERK-positive. The numbers of c-Fos-positive cells within the IB4-stained area and p-ERK-positive cells in laminae I-II were manually counted.

### Western blot analysis of membrane and cytosolic fraction samples from dorsal horn of lumbar spinal cord ipsilateral to formalin-injected hind paw of WT mice

Nine WT mice were used in this study. The mice were lightly anesthetized with halothane, and 20 µl of 5% formalin solution was injected into the left hind paw as in the formalin test described above. The mice were grouped into three, and their spinal cords were sampled 0 min, 30 min, and 90 min after the injection. The dorsal horn of the lumbar spinal cord ipsilateral to the injection site was processed using the method described by Huttner et al. [41] and Balan et al. [42] with slight modifications as follow. A sample of the dorsal horn ipsilateral to the injection site from each mouse was homogenized in HEPES-buffered sucrose [320 mM sucrose, 4 mM HEPES/NaOH (pH 7.3)] supplemented with protease inhibitors (Protease Inhibitor Cocktail, Nacalai Tesque, Kyoto, Japan) using a homogenizer. The homogenate was centrifuged at 1,200 x *g* for 15 min at 4°C to remove cell nuclei and debris. The supernatant was centrifuged at 200,000 x *g* for 120 min at 4°C to obtain cytosolic and membrane fractions. Protein extracts of 11–20 µg (the same weight for each electrophoresis run) were fractionated by SDS-PAGE and fractionated proteins were transferred onto a polyvinylidine difluoride (PVDF) membrane. After blocking with 5% skim milk in PBS containing 0.1% Tween-20, the PVDF membrane was incubated with rabbit anti-SR, rabbit anti-actin, and rabbit anti-TfR antibodies at 4°C overnight. Following the incubation with the appropriate secondary antibody for 40 min at RT, protein bands were detected using an ELC chemiluminescence detection system (Image Quant LAS 400 mini, GE Healthcare, Sweden). The detected protein bands were quantified using Image Quant TL software (GE Healthcare, Sweden).

### Measurement of SR fluorescence intensity in somata of neurons in dorsal horn after formalin injection in WT mice

Six WT mice were used in this study. The mice were lightly anesthetized with halothane, and 20 µl of 5% formalin solution was injected into the left hind paw as in the formalin test described above. The mice were grouped into three and their spinal cords were sampled 0 min, 30 min, and 90 min after the injection. The L4–L5 spinal cords were processed for double immunohistochemical analysis using mouse monoclonal anti-SR and rabbit polyclonal anti-MAP2 antibodies as the primary antibodies. All the sections were counterstained with DAPI for cell nuclear visualization. The immunopositivity for SR and MAP2 in the dorsal horn ipsilateral to the injection site was evaluated. In the set of images obtained from 0 min and 90 min after formalin injection, ten cells that showed the colocalized signals of SR and MAP2 were selected for further analysis. The regions of interest (ROIs) were set as an ellipse (the longest diameter; 7.9±0.3 µm) around the selected cells and the fluorescence intensities of SR and MAP2 within the ROI were measured using the publicly available Java image-processing program ImageJ (National Institute of Health, Bethesda, MD).

### Statistical analyses

All numerical data are presented as means ± S.E.M. The data of the formalin test were examined by two-way repeated measures analysis of variance (RM ANOVA followed by the Tukey-Kramer post-hoc test as indicated. Differences in the numbers of c-Fos- and p-ERK-positive cells in the dorsal horn between the SR-KO and WT mice, and the fluorescence intensity of SR 0 min and 90 min after formalin injection were compared by the unpaired Student's t-test. The SR protein expression levels in the cytosolic and membrane fractions 0 min, 30 min, and 90 min after formalin injection were compared by one-way ANOVA followed by the Tukey-Kramer post-hoc test as indicated. In all comparisons, values of *p*<0.05 were considered significant.

## Results

### Serine racemase in the spinal cord was distributed mainly in lamina II of the dorsal horn

**Figure 1:**
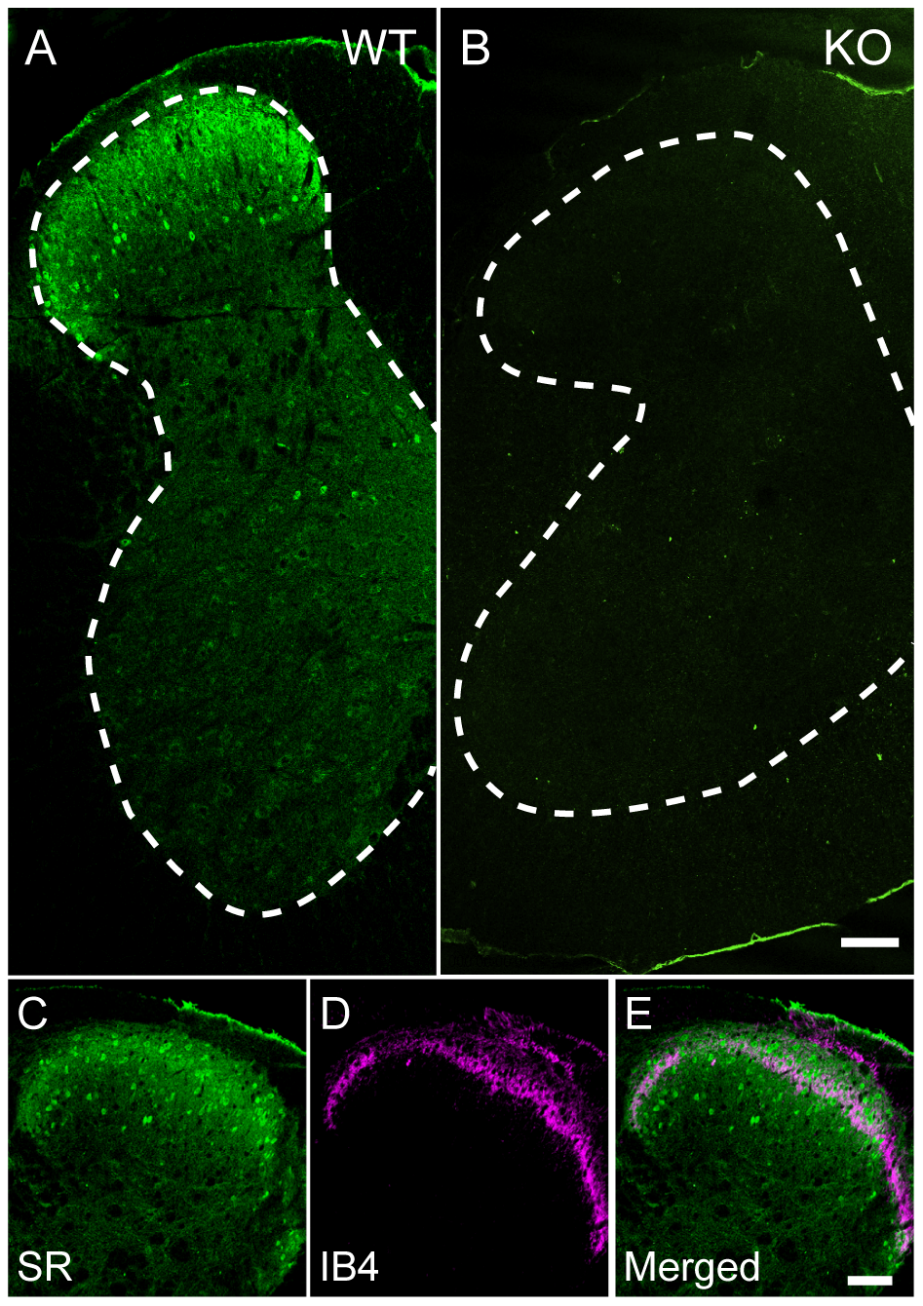
Distribution of immunofluorescence staining of serine racemase (SR) in lumbar spinal cord. (A) SR (green) is mainly distributed in the superficial part of the dorsal horn of the WT mice. (B) No SR signals (green) are detected in the spinal cord of the SR-KO mice. The dorsal part is the upper side. The bar indicates 100 µm in A and B. (C-E) The double immunofluorescence staining of SR (green) and IB4 (magenta), a marker of lamina II of the dorsal horn, indicates that SR is predominantly distributed in lamina II. The bar indicates 75 µm in C-E. The dotted lines in A and B are the outline of the gray matter of the spinal cord.

In the central nervous system, SR is predominantly distributed in neurons of the cerebral cortex, olfactory bulb, olfactory tubercle, hippocampus, and striatum, and is weakly expressed in the cerebellum and brain stem [33]. We first examined the distribution pattern of SR in the spinal cord using SR-KO mice as the negative control. Immunofluorescence staining of SR revealed that SR was distributed mainly in the superficial part of the dorsal horn and was weakly expressed in the cell bodies in the ventral horn (Fig. 1-A). At a higher magnification, SR signals were also found to colocalize with DAPI nuclear staining (Fig. S1). In contrast, no SR signals were detected in the spinal cord of SR-KO mice (Fig. 1-B), suggesting the specificity of SR immunostaining in our study. To determine the localization of SR in the dorsal horn in the Rexed laminae, we performed double fluorescence staining of SR and IB4, which binds nonpeptidergic afferents and terminates on the middle one-third of lamina II [43], [44]. The double fluorescence staining demonstrated that SR in large amounts is distributed in laminae I - III and is concentrated in lamina II of the dorsal horn (Fig. 1-C-E).

### SR-KO mice showed increased pain sensitivity in the second phase of the formalin test

As a potent coagonist of NMDAR, SR in lamina II indicates that D-serine has a role in pain transmission, especially in relation to inflammation. To elucidate the involvement of SR in nociceptive transduction, we conducted the formalin test, a model for inflammatory pain [40], using SR-KO and WT mice.

**Figure 2:**
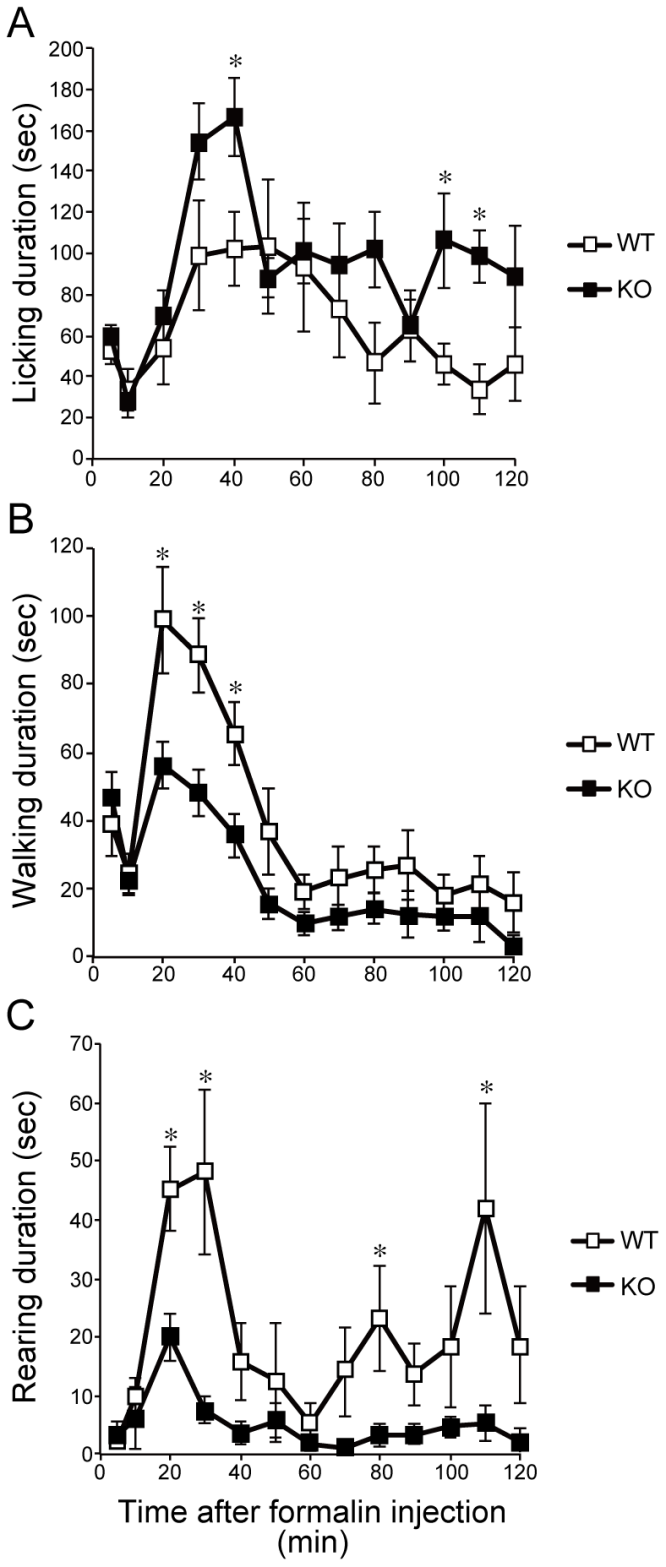
SR-KO mice show significantly enhanced pain behavior during the formalin test. Data from the SR-KO (n = 11, closed square) and WT (n = 9, opened square) mice are the durations of licking (A) of the formalin-injected paw (a pain behavior), and walking (B) and rearing (C) (nonpain behaviors) after formalin injection. Data are collected at 5 min intervals until 10 min after the formalin injection (0 min), then at 10 min intervals until the end of the observation period (120 min). (A) Formalin injected subcutaneously into the hind paw induced biphasic pain responses: an acute temporal but intense licking followed by a short pause (1–10 min; phase 1), and prolonged continuous tonic licking during the observation period (11–120 min; phase 2). There is no significant difference in licking duration between the SR-KO and WT mice in phase 1. However, the SR-KO mice showed significantly enhanced pain behavior in the early (40 min) and late (100 and 110 min) stages of phase 2. (B) In the early stage (20, 30, and 40 min) of phase 2, the WT mice walked significantly longer than the SR-KO mice. There is no significant difference in walking duration between the SR-KO and WT mice in phase 1. (C) The WT mice showed a significant increase in rearing duration in phase 2, especially in the early (20 and 30 min) and late (80 and 110 min) stages. There is no significant difference in rearing duration between the SR-KO and WT mice in phase 1. Values are the means ± SEM. Data were analyzed by two-way RM ANOVA followed by the Tukey-Kramer post-hoc test; * *p*<0.05.

Formalin subcutaneously injected into the hind paw induces a biphasic nociceptive response: immediate and intense responses with a transient quiescent period (phase 1: 1–10 min) followed by a prolonged tonic response (phase 2: 11–120 min) [39], [45]. Phase 1 of the pain behavior (i.e., paw licking) is caused by the direct effect of formalin on nociceptors, which evokes intense but short-duration pain behavior, and phase 2 of the behavior is induced by inflammatory responses caused by formalin [39], [46], [47] and is prevented by NMDA antagonists [14]. Following the formalin injection, both WT and SR-KO mice demonstrated typical biphasic responses. The duration of the pain behavior, that is, licking on the formalin-injected paw, in phase 2 significantly increased in SR-KO mice (two-way RM ANOVA), WT vs SR-KO, F = 8.907, *p*<0.01). There were no significant differences in the intensity of the pain behavior between the SR-KO and WT mice in phase 1. The licking duration in the SR-KO mice was significantly longer than that in the WT mice at 40, 100, and 110 min (Tukey-Kramer post-hoc test, *p*<0.05), (Fig. 2-A).

In the formalin test, we investigated other behaviors that were not signs of pain: walking, rearing, grooming, and crouching. There were significant differences between the WT and SR-KO mice in walking and rearing durations during phase 2 of the formalin test [two-way RM ANOVA, WT vs SR-KO, F = 8.045, *p*<0.05 (walking), F = 9.313, *p*<0.01 (rearing)]. The WT mice walked for a significantly longer time than the SR-KO mice at 20, 30, and 40 min of the observation period (Tukey-Kramer post-hoc test, *p*<0.05), (Fig. 2-B). The rearing durations of the WT mice were significantly longer at 20, 30, 80, and 110 min (Tukey-Kramer post-hoc test, *p*<0.05), (Fig. 2-C). There were no significant differences between the SR-KO and WT mice in grooming and crouching durations in the formalin test [two-way RM ANOVA, WT vs SR-KO, F = 0.61, *p*>0.05 (grooming), F = 0.33, *p*>0.05 (crouching); data not shown].

### The nociceptive cells in the dorsal horn of SR-KO mice showed a markedly high activity during the formalin test

To verify the changes in neuronal activity in the dorsal horn of the spinal cord, we conducted an immunohistochemical study using the antibodies to c-Fos and phosphorylated ERK (p-ERK). c-Fos is the protein encoded by the protooncogene *c-fos* and has been extensively used as the marker of neuronal activity in pain [48], [49], [50]. As an immediate early gene, the transcriptional activation of c-*fos* occurs within minutes after stimulation and the expression level of the protein peaks about 2 h after the induction of gene transcription. The level of formalin-induced c-Fos expression returns to the baseline 8–24 h after formalin injection [50], [51]. p-ERK has recently been commonly used as a nociceptive specific marker in many pain studies [51], [52], [53], [54]. The p-ERK expression in response to noxious stimuli is reported to be transient: rapid onset and peaking at 2–10 min, and returning to the baseline 1–2 h after formalin injection [51], [55]. The high p-ERK expression levels continue along with the pain behaviors [52]. In our formalin test, the pain behavior of WT and SR-KO mice continued to 120 min. Thus, we examined the p-ERK expression level 30 min after the behavioral test.

**Figure 3:**
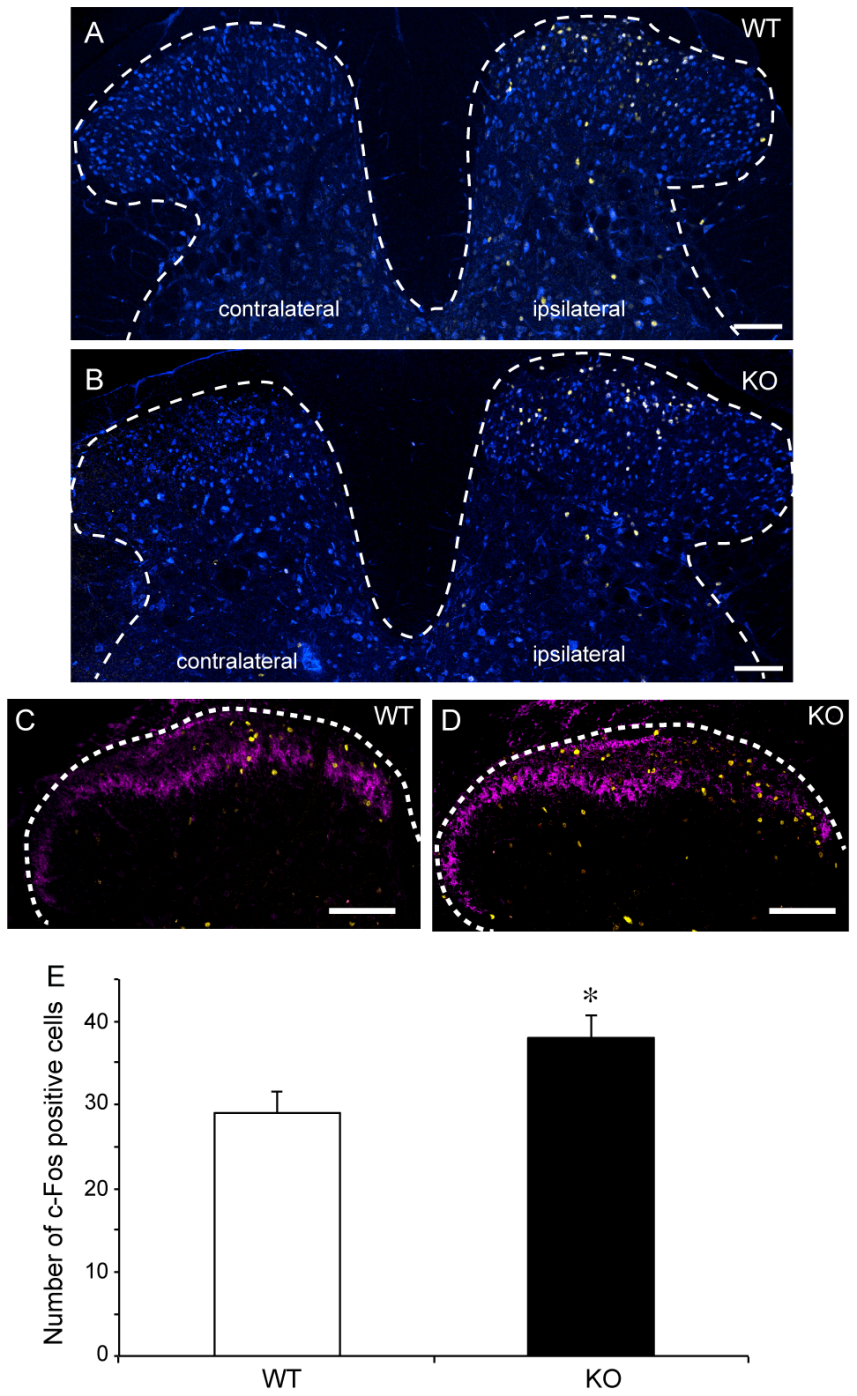
The number of c-Fos-positive neurons in the SR-KO mice significantly increased after the formalin test. (A, B) Immunohistochemical analysis indicates that formalin injected into the left hind paw induced c-Fos protein signals (yellow) in the ipsilateral dorsal horn neurons detected together with NeuN signals (blue) in the WT (A) and SR-KO (B) mice. The c-Fos-positive neurons are mainly distributed in the superficial layers in the dorsal horn. Bars indicate 100 µm. (C, D) Double fluorescence staining with IB4 (magenta) indicates that the c-Fos-positive (yellow) neurons are mainly distributed in laminae I-II of the dorsal horn. Bars indicate 100 µm. Dotted lines in A - D are the outline of the gray matter of the spinal cord. (E) The number of c-Fos-positive cells in laminae I-II significantly increased in SR-KO mice. Values are the means ± SEM. Data are analyzed using the unpaired t-test; * *p*<0.05 (n = 5 for WT and SR-KO mice).

**Figure 4:**
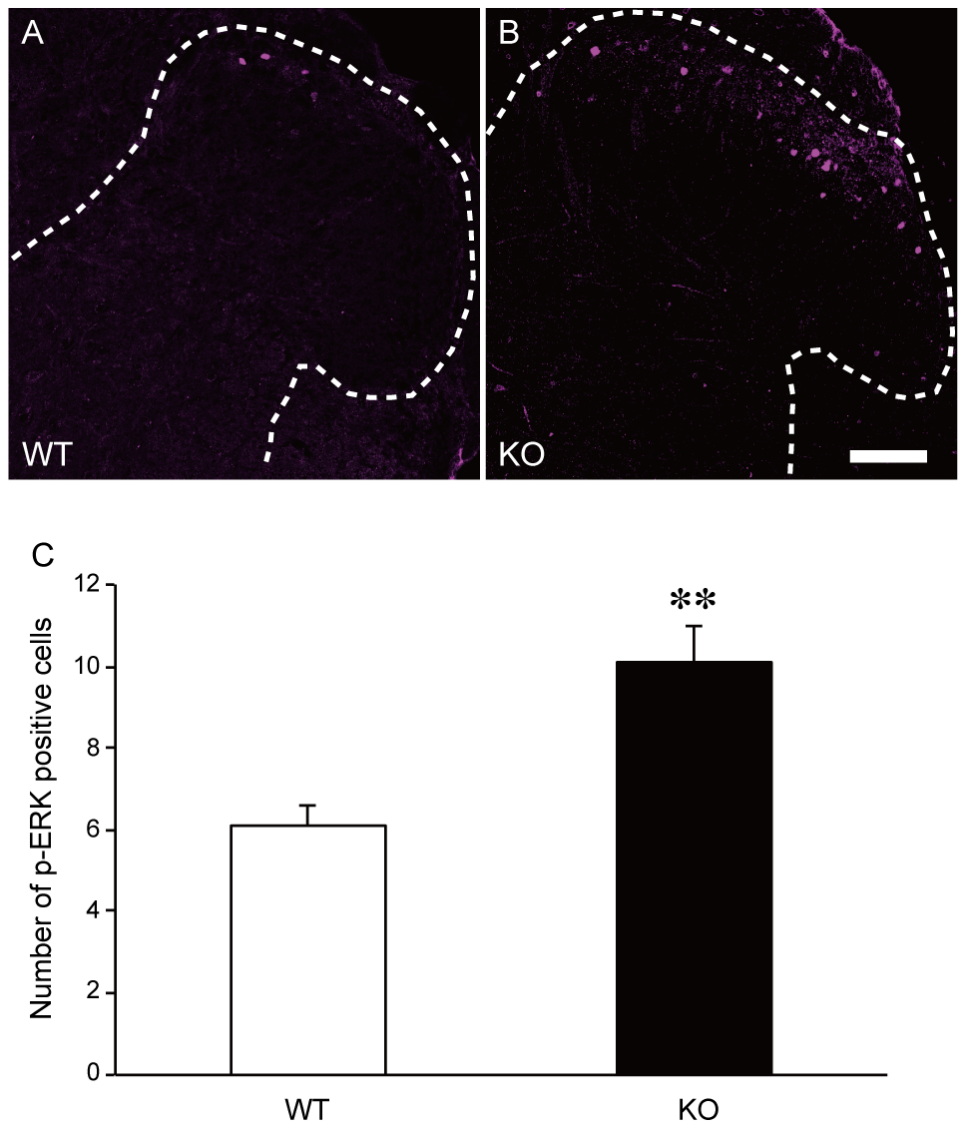
The number of p-ERK-positive neurons in SR-KO mice significantly increased after the formalin test. (A, B) Immunohistochemical analysis indicates that formalin injected into the left hind paw induced p-ERK signals (magenta) at the surface of the ipsilateral dorsal horn of the WT and SR-KO mice. Dotted lines in A and B are the outline of the gray matter of the spinal cord. The bar indicates 75 µm. (C) The number of p-ERK-positive neurons in laminae I–II significantly increased in the SR-KO mice. Values are the means ± SEM. Data are analyzed by the unpaired t-test; ** *p*<0.01 (n = 5 for WT and SR-KO mice).

Figures 3-A and B show the immunofluorescence staining pattern of c-Fos in the L4–L5 spinal cord harvested 30 min after the behavioral test. After the formalin injection into the left hind paw, c-Fos protein signals were observed in the ipsilateral dorsal horn of the spinal cord in the SR-KO and WT mice. Double immunofluorescence staining of NeuN, a neuronal marker, showed the dense distribution of c-Fos-positive neurons in the superficial layers in the dorsal horn of the SR-KO and WT mice. The number of c-Fos-positive neurons in laminae I–II, identified using IB4, was significantly larger in the SR-KO mice (n = 5, unpaired Student's t-test, two-tailed, WT vs SR-KO, *p*<0.05), (Fig. 3-C-E).

p-ERK-positive cells were also observed in the ipsilateral dorsal horn of the spinal cord in SR-KO and WT mice (Fig. 4-A, B). p-ERK was mainly distributed in lamina I–II of the dorsal horn, and the number of the p-ERK-positive neurons identified on the basis of NeuN signals was significantly larger in the SR-KO mice (n = 5, unpaired Student's t-test, two-tailed, WT vs SR-KO, *p*<0.01), (Fig. 4-C).

### Inflammatory stimuli decreased the cytosolic SR expression level in the ipsilateral dorsal horn and induced changes in SR localization in lamina II

Although D-serine is a potent coagonist of NMDAR, the mice lacking SR demonstrated increased sensitivity to inflammatory pain in our study. To clarify the role of SR, we examined the effects of noxious stimuli on SR localization and expression patterns on the basis of the lines of evidence suggesting the feedback inactivation of D-serine synthesis by NMDAR activity, namely, the membrane translocation of SR and the degradation of SR itself [42], [56], [57].

**Figure 5:**
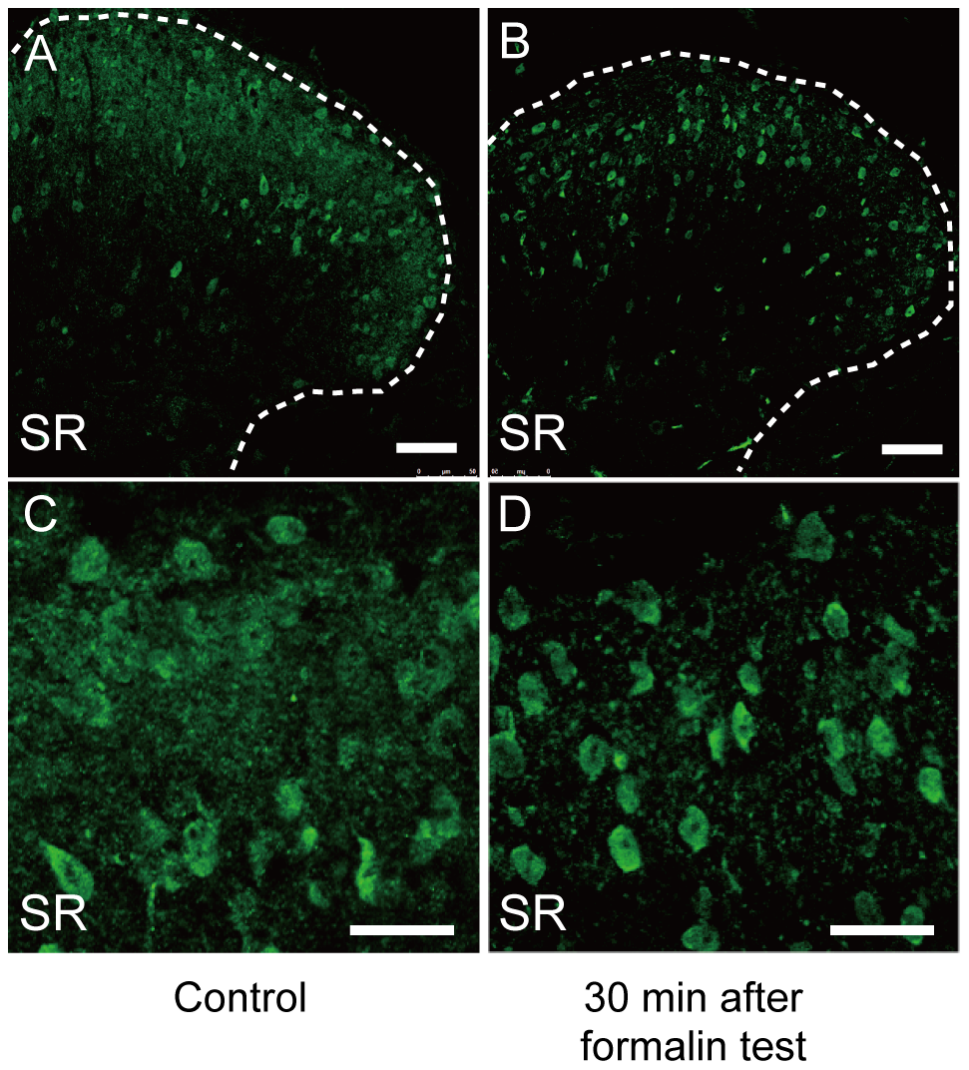
The distribution of SR in the dorsal horn changed after the formalin test. (A, C) Before the formalin injection, SR (green) was uniformly distributed in lamina II of the dorsal horn of the lumbar spinal cord in the WT mice. An enlarged image of lamina II demonstrates a relatively dense distribution of SR signals particularly in cell bodies. Bars indicate 50 µm. (B, D) Thirty minutes after the formalin test, the density of immunofluorescence SR signals in the ipsilateral dorsal horn decreased. A magnified image of lamina II revealed that the signals of SR concentrated in cell bodies (D). Bars indicate 50 µm. Dotted lines in A and B are the outline of the gray matter of the spinal cord.

We first evaluated the SR localization in the dorsal horn of L4–L5 of the lumbar spinal cord from WT mice before and after the formalin test. Immunofluorescence signals of SR were relatively uniformly distributed in lamina II in the control mice (Fig. 5-A). A magnified image of lamina II shows densely distributed SR signals (Fig. 5-C). In contrast, immunopositivity for SR was mainly detected in the cell bodies in lamina II of the ipsilateral dorsal horn 30 min after the formalin test (Fig. 5-B). A magnified image of lamina II reveals that SR signals were concentrated in the cell bodies (Fig. 5-D). These findings suggest that the formalin injection induced the changes in SR localization and expression level.

**Figure 6:**
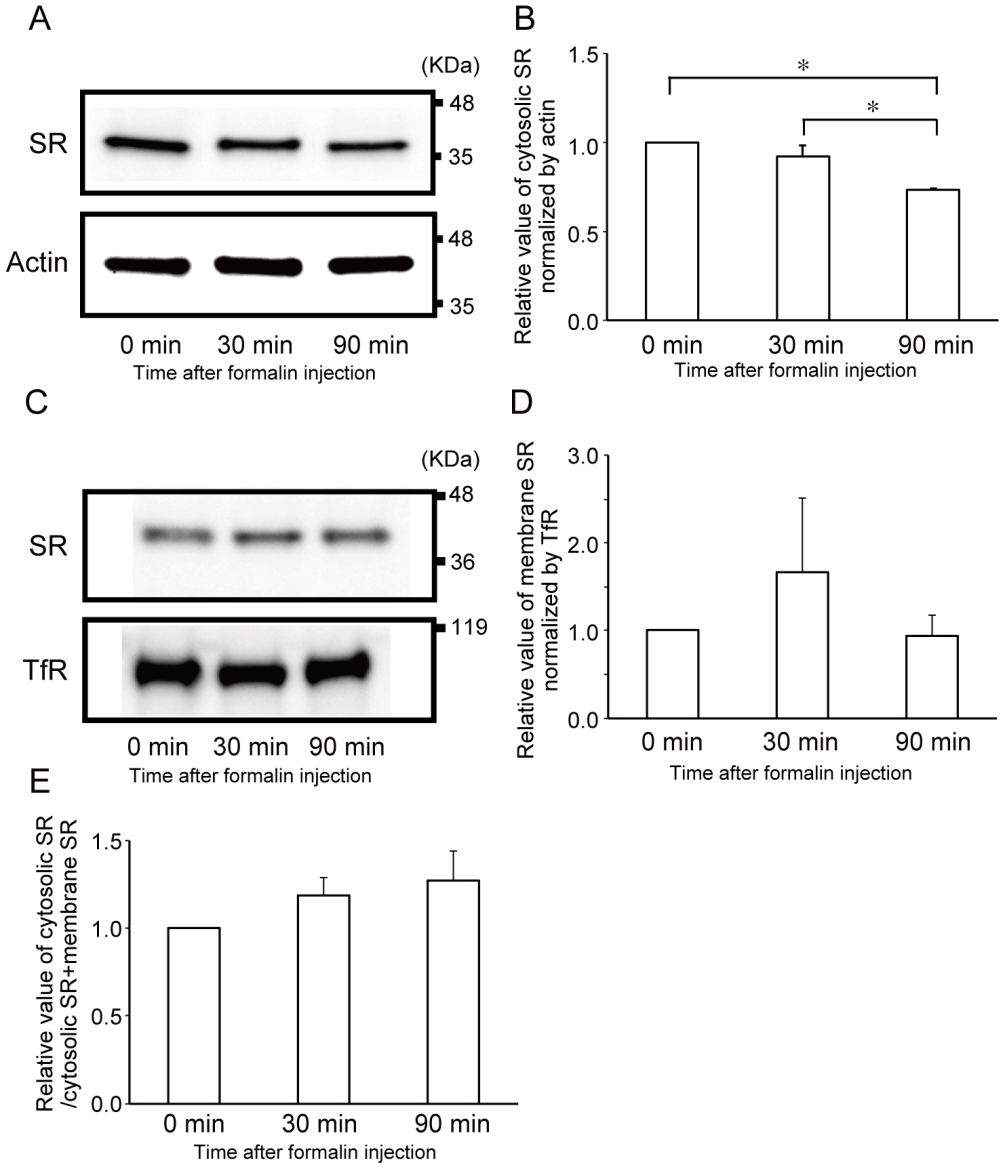
Cytosolic and membrane SR in the ipsilateral dorsal horn changed after the formalin test. Western blot analysis of SR in cytosolic and membrane fraction samples from ipsilateral dorsal horn of lumbar spinal cord in WT mice was performed 0 min, 30 min, and 90 min after formalin injection. (A) Western blot analysis of the cytosolic fraction revealed the decrease in SR protein expression level after formalin injection. (B) The relative SR expression level 90 min after the injection significantly decreased from the control level. The expression level of the SR protein was normalized to that of actin. (C) SR expression levels in membrane fraction before and after formalin injection. (D) Relative SR expression level after formalin injection. The expression level of the SR protein was normalized to that of the transferrin receptor protein (TfR). (E) Relative change in SR expression level in membrane fraction to those in cytosolic and membrane fractions after formalin injection. The positions of protein size markers are indicated on the right side (A, C). Values are the means ± SEM. Data are analyzed by one-way ANOVA followed by Tukey-Kramer post-hoc test; * *p*<0.05 (n = 3 for WT and SR-KO mice).

To examine the temporal changes in the level of SR expression after the formalin injection, the ipsilateral dorsal horn of the lumbar spinal cord in WT mice was harvested 0 min, 30 min, and 90 min after the formalin injection and the cytosolic and membrane fraction samples were prepared and analyzed by western blotting. After the formalin injection, the SR expression level in the cytosolic fraction significantly decreased only 90 min after the injection (one-way ANOVA, F = 14.847, *p*<0.01, Tukey-Kramer post-hoc test, *p*<0.05, Fig. 6-A, B). The SR expression level in the membrane fraction tended to increase after the formalin injection, but there is no significant difference in membrane SR level among the groups examined at 0, 30, and 90 min (one-way ANOVA, F = 1.89, *p*>0.05, Fig. 6-C, D). The expression level of the membrane SR relative to that of the total SR (cytosolic and membrane) showed no significant difference among the groups examined at 0 min, 30 min, and 90 min (one-way ANOVA, F = 1.4235, *p*>0.05, Fig. 6-E).

**Figure 7:**
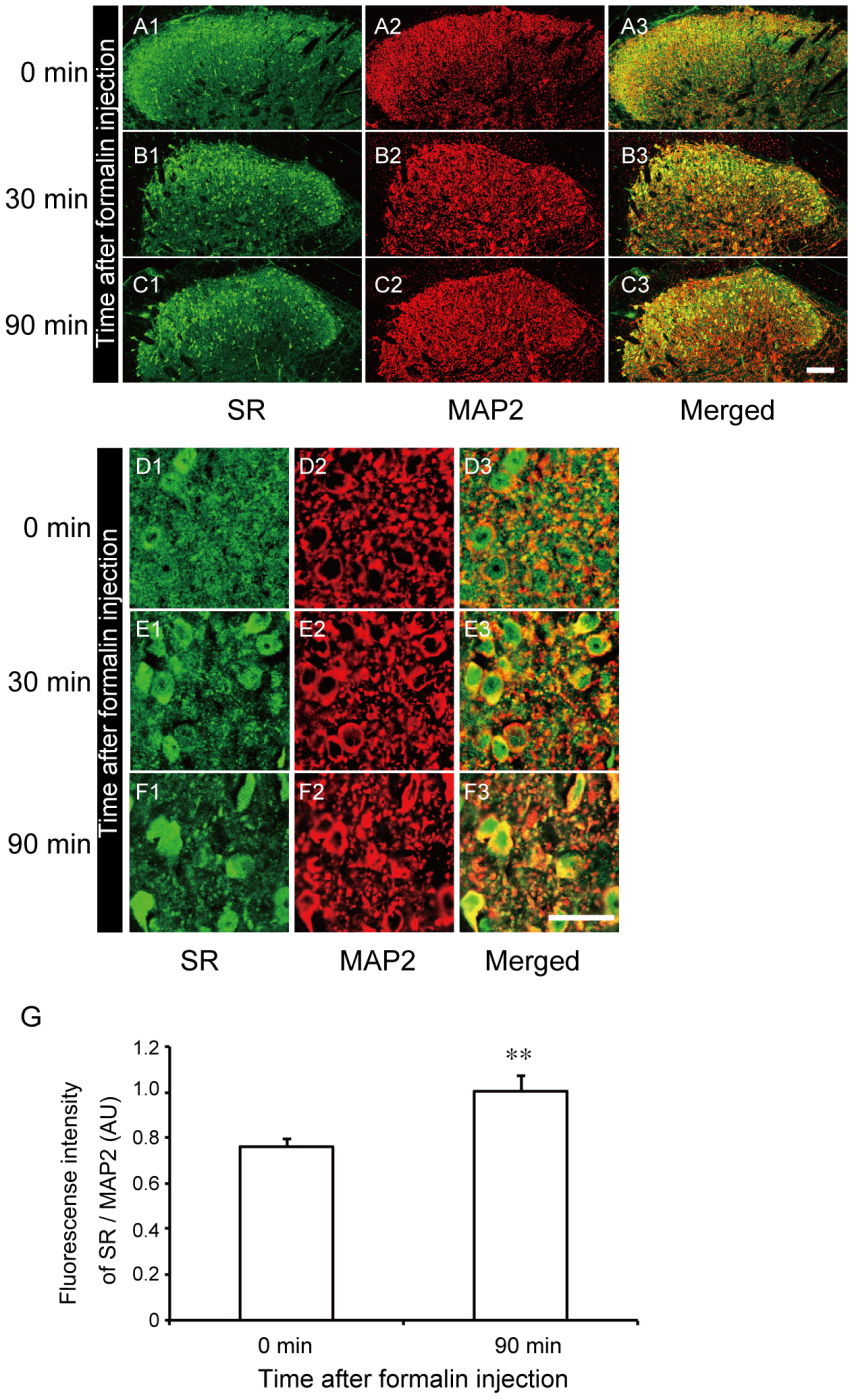
Immunofluorescence localization of SR in ipsilateral dorsal horn changes after formalin injection. (A-C) In WT mice, SR (green) is predominantly distributed in lamina II of the dorsal horn before formalin injection (0 min), and its signal density decreases over time after the injection (A1-C1). Double immunofluorescence staining of SR (green) and microtubule-associated protein 2 (MAP2) (red, A2-C2) indicates that SR and MAP2 colocalize in lamina II of the dorsal horn. The merged signals of SR and MAP2 are broadly distributed in the control (A3-C3). The bar indicates 75 µm in A - C. (D-F) Magnified image of areas of SR and MAP2 colocalization before and after formalin injection. Signals of SR and MAP2 are merged and densely distributed before formalin injection (D3). After formalin injection, the density of merged signals of SR and MAP2 increased in cell bodies and decreased around the cell bodies (E3, F3). The bar indicates 25 µm in D-F. (G) The relative fluorescence intensity of SR in cell bodies 90 min after the formalin injection significantly increased from the control level. The fluorescence intensity of the SR protein was normalized to that of MAP2. AU, arbitrary units. Values are the means ± SEM. Data are analyzed by the unpaired t-test; ** *p*<0.01 (n = 10 for 0 min and 90 min).

For the detailed analysis of SR localization after formalin injection, immunohistochemical analysis was carried out using the sections from the L4–L5 of the lumbar spinal cord harvested 0 min, 30 min, and 90 min after formalin injection. Immunofluorescence staining of SR indicated that the observed SR signals at lamina II of the dorsal horn at 0 min became weak 30 min after the formalin injection (Fig. 7-A1-C1, D1-F1). Double immunofluorescence staining with MAP2 (Fig. 7-A2-C2, D2-F2), the marker of neuronal somata and dendrites, revealed the colocalization of SR and MAP2 (Fig. 7-A3-C3). A magnified image of lamina II revealed that SR signals uniformly colocalized with MAP2 signals before the formalin injection, and the area of colocalization of SR and MAP2 signals decreased 30 min and 90 min after the formalin injection. Most of the merged areas were observed in the cell body after the formalin injection (Fig. 7-D3-F3). To compare the fluorescence intensity of SR in the cell body before and after the formalin injection, the fluorescence intensity of SR was normalized with MAP2 signal intensity. We found that the signal of SR in the soma 90 min after formalin injection significantly increased (n = 10, unpaired Student t-test, 0 min vs 90 min, *p*<0.01, Fig. 7-G).

### Effect of exogenous D-serine on pain behavior in WT and SR-KO mice

**Figure 8:**
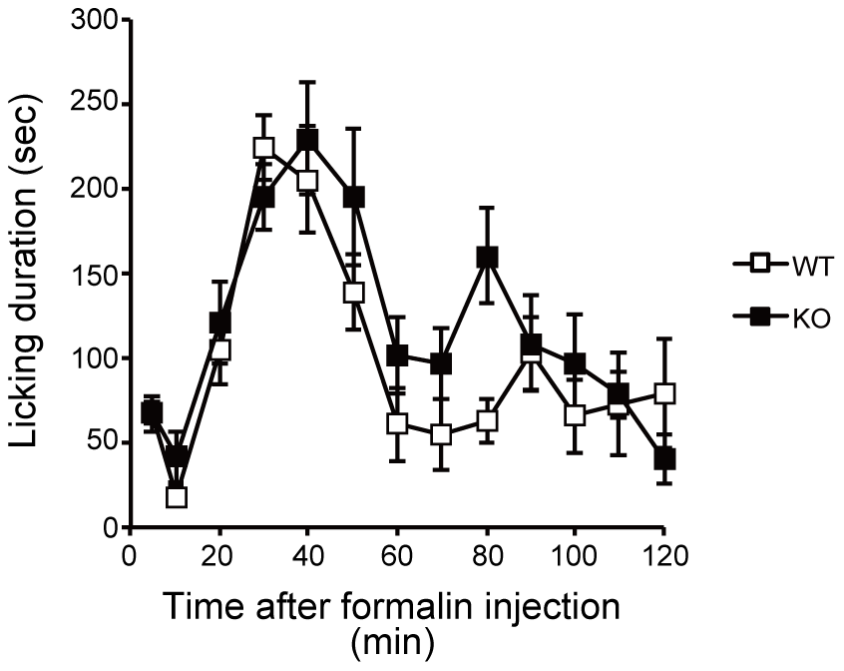
Effect of exogenous D-serine on pain behavior in WT and SR-KO mice. Data from the SR-KO (n = 10, closed square) and WT (n = 9, opened square) mice are the durations of licking of formalin-injected paw (a pain behavior) after formalin injection. Data are collected at 5 min intervals until 10 min after the formalin injection (0 min), then at 10 min intervals until the end of the observation period (120 min). Formalin injected subcutaneously into the hind paw induced biphasic pain responses. There is no significant difference in licking duration between the SR-KO and WT mice during the formalin test. Values are the means ± SEM. Data were analyzed by two-way RM ANOVA.

To evaluate whether the D-serine deficit in SR-KO mice is responsible for the pain hypersensitivity, we conducted the formalin test after the administration of exogenous D-serine. Because the administration of 10 mM D-serine in drinking water for one week significantly increased the spinal D-serine level in SR-KO mice [58], we conducted the formalin test one week after oral administration of 10 mM D-serine. Formalin subcutaneously injected into the hind paw induced typical biphasic responses in WT and SR-KO mice. There were statistically no significant differences in the intensity of the pain behavior between SR-KO and WT mice during the formalin test (two-way RM ANOVA, F = 4.15, *p* = 0.06), (Fig. 8).

## Discussion

The aim of this study was to identify the role of SR in nociceptive transmission in the dorsal horn of the spinal cord using the mutant mice lacking SR. The main findings of this study were as follows: 1) SR was mainly distributed in lamina II of the dorsal horn of the spinal cord in the WT mice, 2) the SR-KO mice showed increased sensitivity to inflammatory pain induced by formalin injected subcutaneously, 3) the expression levels of the c-Fos and p-ERK proteins, the molecular markers of pain, significantly increased in laminae I-II of the ipsilateral dorsal horn neurons in SR-KO mice after the formalin test, 4) the immunopositivity level of SR decreased and the SR distribution pattern changed from being broad to being concentrated in cell bodies in lamina II after the formalin test, 5) the expression level of the SR protein significantly decreased in the cytosolic fraction 90 min after the noxious stimulation with formalin, 6) SR colocalized with MAP2, a marker of neuronal somata and dendrites, and the area of colocalization changed from being broad to being concentrated in cell bodies in lamina II after formalin stimuli, 7) D-serine administration for one week cancelled the difference in pain behaviors between WT and SR-KO mice. These findings indicate that SR in lamina II of the dorsal horn has the novel inhibitory role in pain transmission via modulation of SR localization and expression.

### SR expression pattern in spinal cord

D-Serine in the brain exists at high levels in areas where NMDAR, particularly the NR2B-subunit-containing receptors, are abundant [24] and regulates NMDAR as a potent and dominant coagonist over another coagonist, glycine [5], [59]. Because the mutant mice without the SR protein show a marked decrease in the expression level of D-serine (10–20% of WT mice) in the brain [33], [60], [61] and because D-serine distribution closely correlates with that of SR [2], [62], SR is a predominant enzyme for D-serine production [63]. Accordingly, SR has been considered an important enzyme for effective neurotransmission and synaptic plasticity via NMDAR activation. Although its expression level is low, D-serine exists in the spinal cord and is most likely produced from SR [1], [58], [64]. In our study, SR was found to distribute mainly in sensory neurons in lamina II of the dorsal horn. Nagy et al. immunohistochemically showed that among NMDAR subunits, the NR2B subunit is mainly distributed in lamina II of the dorsal horn [18]. This coexpression of SR and the NR2B subunit in lamina II suggests that D-serine has an important contribution to sensory transmission, particularly pain sensation, through NMDAR activation.

### Formalin test

To demonstrate the involvement of SR in the pain pathway, we examined the SR-KO and WT mice by the formalin test, which induces inflammation and C-fiber-specific responses resulting in the central sensitization that requires NMDAR activation [14], [39], [65]. We hypothesized that if D-serine in the dorsal horn is important for central sensitization, the SR-KO mice should show attenuated pain behavior in the second phase of the formalin test. Contrary to the hypothesis, the SR-KO mice showed significantly enhanced pain behavior in the second phase. The nonpain behaviors, such as walking and rearing, were reduced in the SR-KO mice. Altogether, these findings confirm that SR-KO mice have increased sensitivity to inflammatory pain.

Hypersensitivity to inflammatory pain seen in the SR-KO mice is further supported by the increased expression levels of the c-Fos and p-ERK proteins in laminae I-II of the ipsilateral dorsal horn. Thirty minutes after the formalin test, the time when the expression level of c-Fos normally peaks and that of p-ERK continues, the numbers of c-Fos- and p-ERK-immunopositive cells were significantly larger in the SR-KO mice; therefore, the neuronal activity for nociceptive transmission is considered higher and the neuronal activation longer in the SR-KO mice.

Although it has been reported that licking behavior itself increases the c-Fos expression level [66] and decreases the p-ERK expression level [67], the effect of licking on c-Fos and p-ERK expression levels was excluded in our study because not only the expression level of c-Fos but also that of p-ERK increased in the SR-KO mice. The intense and prolonged neuronal activation in response to the formalin injection reflects the prolonged pain behavior seen in the SR-KO mice, which reinforce the idea that the SR-KO mice have increased sensitivity to inflammatory pain.

### Inhibitory modulation by SR in pain pathway

Our results clearly show that SR-KO mice have increased sensitivity to inflammatory pain, indicating that the presence of SR and possibly D-serine is required for the proper inhibitory modulation in the pain pathway. There are lines of evidence showing that D-serine production is regulated by the activation and inactivation of SR. Balan et al. reported that SR translocates from the cytosol to the membrane and is inactivated by palmitoylation to inhibit D-serine synthesis after NMDAR activation [42]. Dumin et al. reported that the D-serine level is modulated via ubiquitin-dependent degradation of SR [56]. In our study, SR was found to colocalize with MAP2 and its distribution pattern changed from being broad to being concentrated in cell bodies after the formalin injection in the WT mice. On the other hand, the expression level of the SR protein significantly decreased in the cytosolic fraction. These findings indicate that the expression level of SR decreased and SR translocated into cell bodies after the formalin stimulation, suggesting that the feedback inactivation of D-serine synthesis was induced by formalin. That is, the inflammatory pain stimulation by formalin in peripheral tissues is transmitted into lamina II of the dorsal horn via C-fibers and activates NMDAR; consequently, the translocation and degradation of SR, and the decrease in cytosolic SR expression level will be induced for the inactivation of D-serine synthesis to initiate the analgesic pathway. The results of our formalin test after oral administration of D-serine for one week support this hypothesis. Miyoshi et al. reported that oral administration of D-serine significantly increases the spinal D-serine level in SR-KO mice [58]. After oral administration of D-serine, the pain behavior in phase 2 of the formalin test was enhanced in both WT and SR-KO mice to the same degree. This suggests that excess amount of D-serine in the spinal cord caused by D-serine administration cancelled the SR feedback inactivation system, which attenuates the excessive NMDAR activation.

In SR-KO mice, the SR-regulated inhibitory control is absent and glycine is the only coagonist driving NMDAR. Because D-serine activates NMDAR more efficiently than glycine does [5], it is presumable that the NMDAR barrage induced by formalin was more intense in the WT mice, at least at the beginning of the inflammatory pain transmission. Nevertheless, the SR-KO mice showed hypersensitivity to inflammatory pain. It is possible that an as yet compensatory mechanism by which the level of glycine increases in the spinal cord exists in SR-KO mice. Furthermore, Basu et al. examined the decay kinetics of NMDAR-mediated excitatory postsynaptic current recording from hippocampal cells of SR-KO mice and suggested an enhanced contribution of NR2B-containing NMDAR in the juvenile stage of SR-KO mice [60]. Hence, because the NR2B subunit is involved in pain transmission [19], [20], [21], [22], [23], an enhanced contribution of NR2B-containing NMDAR could be another possible reason for the enhanced pain transmission seen in SR-KO mice.

Our results indicate the following novel hypothetical analgesic mechanism regulated by SR. When noxious stimuli are transmitted to the dorsal horn, SR residing broadly in lamina II of the dorsal horn translocates to the cell body resulting in the inactivation of D-serine production. At the same time, the expression level of SR in the cytosol decreases, which leads to decreased D-serine production, which in turn attenuates NMDAR activation and consequently alleviates nociceptive sensation.

## Conclusions

SR, which converts L-serine to the potent coagonist D-serine for NMDAR, is distributed in areas where NMDAR, especially its NR2B subunit, is abundant. Under physiological condition, D-serine plays an important role in synaptic plasticity enhancing neurotransmission. In this study, we illustrated the inhibitory modulation regulated by SR in the spinal pain pathway. This is a novel mechanism of the analgesic pathway for inflammatory pain. With the effective agents blocking D-serine production, this inhibitory pathway may provide future analgesic strategies for controlling inflammatory pain.

## Supporting Information

Figure S1 **Immunofluorescence localization of SR and DAPI in single optical section in dorsal horn of lumbar spinal cord.** (A-C) In WT mice, SR (green) is predominantly distributed in lamina II of the dorsal horn without formalin injection (A). Double fluorescence staining of SR (green) and DAPI (blue, B) indicates that SR signals colocalize with DAPI nuclear staining (C). The bar indicates 25 µm in A - C.
(TIF)
