# Supplementary figures and images for: Increased Sensitivity to Inflammatory Pain Induced by Subcutaneous Formalin Injection in Serine Racemase Knock-Out Mice
Source: PLoS One. 2014 Aug 18;9(8):e105282. doi: 10.1371/journal.pone.0105282 (PMC4136830; doi:10.1371/journal.pone.0105282)

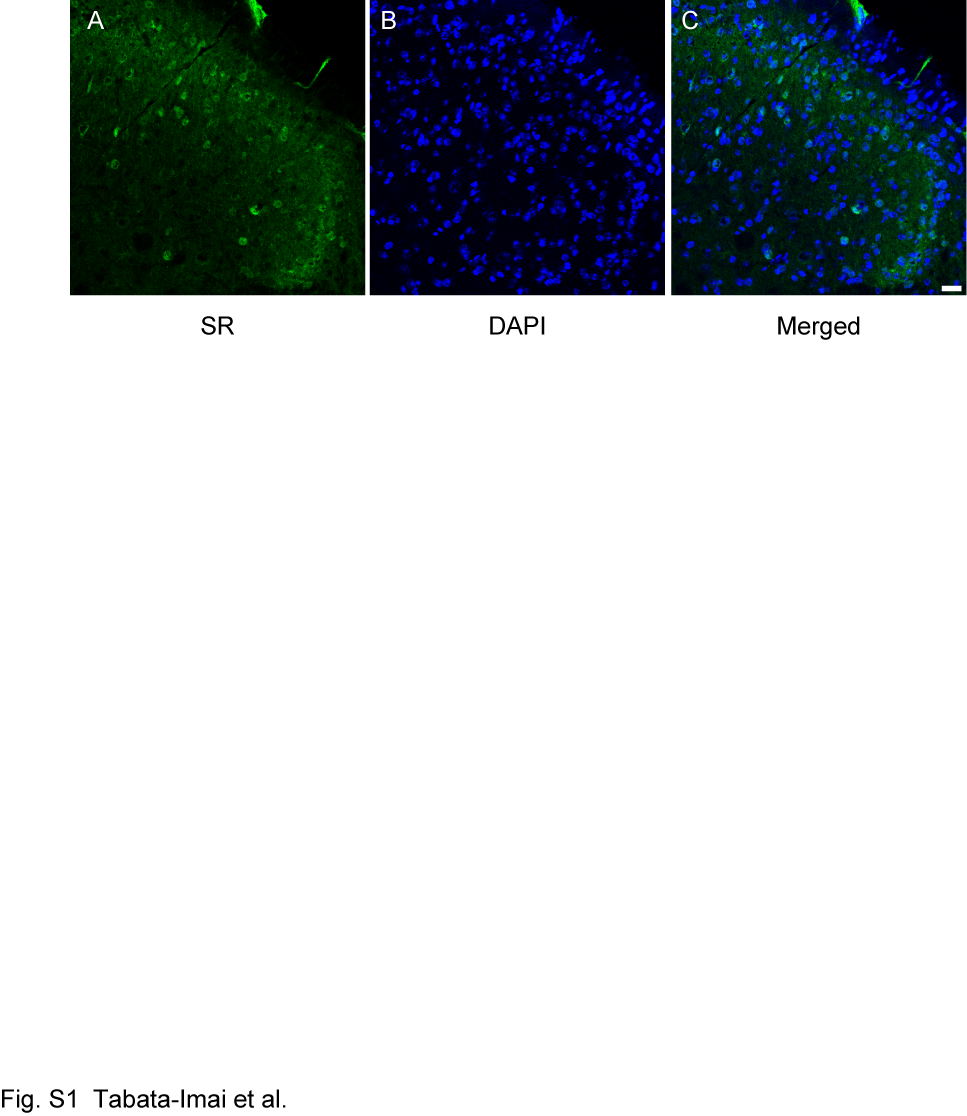

Supplement: Figure S1 — Immunofluorescence localization of SR and DAPI in single optical section in dorsal horn of lumbar spinal cord. (A-C) In WT mice, SR (green) is predominantly distributed in lamina II of the dorsal horn without formalin injection (A). Double fluorescence staining of SR (green) and DAPI (blue, B) indicates that SR signals colocalize with DAPI nuclear staining (C). The bar indicates 25 µm in A - C. (TIF) [file pone.0105282.s001.tif]
